# Understanding Calcium-Dependent Conformational Changes in S100A1 Protein: A Combination of Molecular Dynamics and Gene Expression Study in Skeletal Muscle

**DOI:** 10.3390/cells9010181

**Published:** 2020-01-10

**Authors:** Navaneet Chaturvedi, Khurshid Ahmad, Brijesh Singh Yadav, Eun Ju Lee, Subash Chandra Sonkar, Ninoslav Marina, Inho Choi

**Affiliations:** 1 Department of Bioengineering, University of Information Science and Technology, St. Paul The Apostle, Ohrid-6000, North Macedonia; brijeshbioinfo@gmail.com (B.S.Y.); rector@uist.edu.mk (N.M.); 2 Department of Medical Biotechnology, Yeungnam University, Gyeongsan 38541, Korea; ahmadkhursheed2008@gmail.com (K.A.); gorapadoc0315@hanmail.net (E.J.L.); 3 Department of Obstetrics and Gynaecology, Vardhman Mahavir Medical College and Safdarjang Hospital, New Delhi-110029, India; drscsonkar@gmail.com

**Keywords:** calcium-binding protein, protein-protein interaction, entry of free calcium ions, molecular dynamics, cell culture

## Abstract

The S100A1 protein, involved in various physiological activities through the binding of calcium ions (Ca^2+^), participates in several protein-protein interaction (PPI) events after Ca^2+^-dependent activation. The present work investigates Ca^2+^-dependent conformational changes in the helix-EF hand-helix using the molecular dynamics (MD) simulation approach that facilitates the understanding of Ca^2+^-dependent structural and dynamic distinctions between the apo and holo forms of the protein. Furthermore, the process of ion binding by inserting Ca^2+^ into the bulk of the apo structure was simulated by molecular dynamics. Expectations of the simulation were demonstrated using cluster analysis and a variety of structural metrics, such as interhelical angle estimation, solvent accessible surface area, hydrogen bond analysis, and contact analysis. Ca^2+^ triggered a rise in the interhelical angles of S100A1 on the binding site and solvent accessible surface area. Significant configurational regulations were observed in the holo protein. The findings would contribute to understanding the molecular basis of the association of Ca^2+^ with the S100A1 protein, which may be an appropriate study to understand the Ca^2+^-mediated conformational changes in the protein target. In addition, we investigated the expression profile of S100A1 in myoblast differentiation and muscle regeneration. These data showed that S100A1 is expressed in skeletal muscles. However, the expression decreases with time during the process of myoblast differentiation.

## 1. Introduction

S100 proteins belong to the calcium ion (Ca^2+^)-binding proteins family engaged in Ca^2+^ regulation in various tissues and organs. Many of the S100 proteins are significantly expressed in the cardiac tissue, and has been correlated with heart failure. S100A1 is adequately expressed in the cardiac muscles, skeletal muscle fibers, and brain [1]. S100A1 is especially found in the heart and in lower concentrations in the skeletal muscles in both animals and humans [2]. S100A1 expression increases progressively during cardiac development in mice and grasps a plateau in the postnatal state in the ventricular myocardium [3]. S100A1 controls cardiac Ca^2+^ cycling, both the release and reuptake, and it is also essential for cardiac muscle contractility and in the regulation of mitochondrial metabolism [2,4]. Importantly, the S100A1 protein is expressively downregulated in human end-stage heart failure, interpreting S100A1 and role of Ca^2+^ appropriate targets for cardiac gene therapy [5,6].

**Figure 1:**
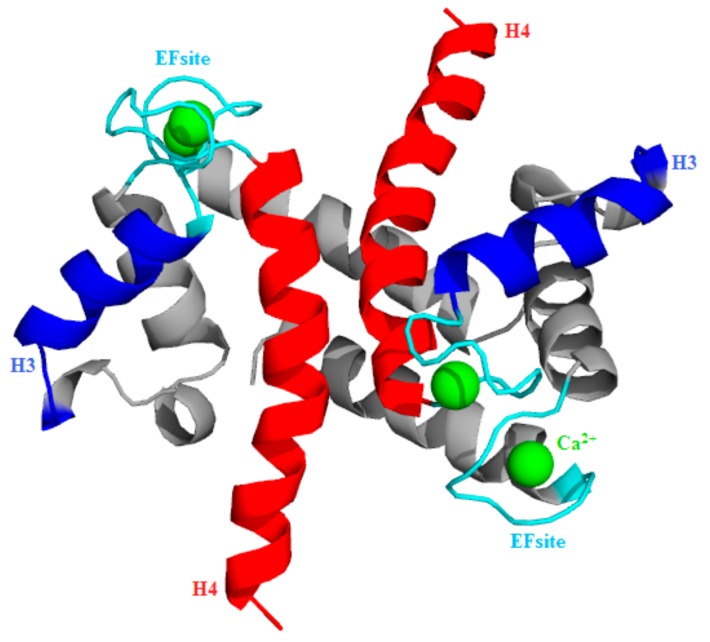
Visualization of homo-subunit S100A1 protein structure (PDB-ID: 2LP3). H3-EF site-H4 topology is depicted, using, respectively, the blue-cyan-red color code pattern. Ca^2+^ (green balls) are positioned on the EF site loop (cyan).

The S100A1 protein typically functions as a homodimer where each monomer contains two EFsite Ca^2+^-binding motifs and each motif has the capacity to bind a single Ca^2+^ [7]. Thus, the monomer contributes two EF site domains, which play a characteristic role in Ca^2+^ binding [7,8]. The first EF site comprises 14 residues and the second site comprises 12 residues [9,10]. The S100A1 subunit loaded with two Ca^2+^ causes a conformational change from an initial to a final state characterized by the exposure of hydrophobic residues between the H3 and H4 domains [11,12]. The opening of H3 and H4 domains describes hydrophobic patches and, consequently, promotes PPI [13], similar to that observed with the calmodulin protein (Figure 1) [14,15,16,17,18].

This study involves molecular dynamics (MD) simulations of S100A1 for examining the characteristics of the apo and holo states. The study also focuses on the entry of free Ca^2+^ into the bulk of the apo state to observe conformational changes in forming the holo state. In addition, Ca^2+^- binding residues were also considered to examine the binding event and formation of new bonds through water. The results support prior molecular simulation and experimental studies [12,19], which suggests that EF site saturation with Ca^2+^ is required for stabilization of the unlocked state. In this study, unbiased MD simulations were performed for each level of complexity to understand the novel behavior of Ca^2+^ bound to the S100A1 protein.

In previous studies, our group and other researchers have explored the importance of Ca^2+^ signaling and that of the associated proteins in skeletal muscle functions [20,21,22]. The contractile properties of skeletal muscles are largely reliant on the protein expression involved in the signaling of Ca^2+^ as this is one of the main signaling and regulatory molecules of myofibers [20]. The multinucleated cells that are the fundamental units of skeletal muscles formed during the differentiation process through the fusion of myoblast cells [21,23].

The damaged skeletal muscles exhibit an extraordinary ability for repair. New muscle fibers are regenerated immediately after injury using complex physiological processes [24]. To explore the function of S100A in muscle regeneration, cardiotoxine (CTX), which is generally used to induce muscle injury, was injected in the muscles of mice. The S100A protein expression during the muscle regeneration process was determined, and found no significant effect of this protein in muscle regeneration. Additionally, we have analyzed the S100A1 expression profile during myoblast differentiation. To explicate the function of S100A1, myoblast cells were cultured in myogenic differentiation media, and mRNA and protein expressions were analyzed at different time points in the differentiated cells.

## 2. Materials and Methods

### 2.1. System Building

The S100A1 protein was simulated using the coordinates retrieved from the Protein Data Bank for the apo and holo states (PDB ID: 2LLU and 2LP3, respectively) [25,26]. Gromacs 4.6.7 suite of the package was employed to carry out MD simulations [27] with the 53a6 parameter set of the Gromos96 force field [28]. We used improved non-bonded parameters for Ca^2+^, as previously recommended, i.e., C^6^ = 2.0e-3 KJ mol^−1^ nm^6^, C^12^ = 1.67e-6 KJ mol^−1^ nm^12^ [29].

### 2.2. Simulation Details

The simulation was executed as described previously by our group [30,31]. The protein was occupied in a dodecahedron box filled with the simple point charge (SPC216) [32] water model that extended up to at least 1.2 nm from the edge of the protein. Na^+^ and Cl^−^ ions were added to neutralize the system up to a concentration of 100 mM. After the solvation and neutralization steps, each system went through an energy minimization process to eliminate steric clashes between atoms. The equilibrium with the solvent started with a 200 ps-long position-controlled simulation under a relentless force of 1000 kJ mol^−1^nm^−1^ at 300 K. Then, the system proceeded for simulation without restrains for 2000 ps, which allows its final equilibrium.

### 2.3. Trajectory Analysis

Since the holo-S100A1 system contains 4 Ca^2+^, an experiment for determining the entry of free Ca^2+^ was set up by adding the same number of Ca^2+^ into the water box of the apo-S100A1 system. Ions were first added into a water solvent box and were left to travel freely. The initial position of Ca^2+^ in the water box was selected randomly. Subsequently, the remaining charges in the system were balanced by adding Na^+^ and Cl^−^ ions. All the algorithms and parameters were considered similar to the holo-S100A1 system. The approximation of conformational changes during the simulation was characterized by interhelical angle estimations, solvent accessible surface area (SASA) determination, H-bond calculations, and contact map analysis. Interhelical angles have been generally employed to compare the transitions in apo-S100A1 and holo-S100A1 proteins during simulations. The average angle values between the common residue of H3, the EF site, and the H4 region of both sides were calculated separately. Trajectories obtained (Table 1) from various simulations were further analyzed. A set of Perl programs was scripted for an approximation of contact analysis within the trajectory. For graph visualization, XMGRACE (http://plasmagate.weizmann.ac.il) was used. Visual molecular dynamics (VMD) [33] and PyMOL packages were used for visualization and system inspections.

### 2.4. CTX-Induced Muscle Regeneration

Muscle injury experiments, for investigating the role of S100A1 in muscle regeneration, were executed as described before [34]. In total, 100 µL of CTX (10 mM) was injected into the left gastrocnemius (GAS) muscles of C57BL/6 mice, and phosphate-buffered saline was injected into the right GAS muscles as a control. Muscle tissues were collected 7 days after the injection. All treatments were administered using the standard protocol permitted by the Institutional Animal Care and Use Committee, Yeungnam University (permit number: YUMC-AEC2015-006).

### 2.5. C2C12 Cell Culture

C2C12 cells (murine myoblast; Korean Cell Line Bank, Seoul, Korea) were cultured in DMEM (HyClone Laboratories, UT, USA), which added 10% FBS (fetal bovine serum) and 1% P/S (Penicillin/Streptomycin, ThermoFisher Scientific, MA, USA) in a humidified CO_2_ incubator at 37 °C. For differentiation experiments, cells were cultured with DMEM + 2% FBS + 1% P/S for 0, 2, 4, and 6 days.

### 2.6. RNA Isolation, cDNA Synthesis, and Real-Time RT-PCR

RNA was isolated with a Trizol reagent (ThermoFisher Scientific) following the manufacturer’s protocols. Two micrograms of RNA in 20 µL of the reaction mixture was used for cDNA synthesis with a random hexamer, and, consequently, reverse-transcribed at 25 °C for 10 min, 37 °C for 120 min, and 85 °C for 5 min. Two microliters cDNA and 2 µL of S100A primer (10 pmole, forward: ccatggagaccctcatcaat, reverse: ttgaagtccacttccccatc) were analyzed using a 7500 real-time PCR system. A Power SYBR Green PCR Master Mix is applied as the fluorescence source.

### 2.7. Western Blot

Proteins at the different time points were isolated using RIPA buffer supplemented with a cocktail of protease inhibitors. Forty microgram of proteins were electrophoresed in 12% polyacrylamide gel and then transferred to a membrane (EMS–Millipore, Billerica, MA, USA). The blot was incubated with 1% skim milk for blocking non-specific binding and then incubated overnight with S100A (1:500) or a β-actin (1:1000) antibody at 4 °C. The blot was washed and incubated with a horse radish peroxidase (HRP)-conjugated secondary antibody for 1 h at 25 °C and then developed with a Super Signal West Pico Chemiluminescent Substrate (ThermoFisher Scientific).

### 2.8. Immunocytochemistry

Cells obtained at the different time point were fixed with 4% formaldehyde (Sigma Aldrich, St. Louis, MO, USA) and permeabilized with 0.2% Triton X and then blocked with 1% normal goat serum (KPL, MD, USA). The cells were incubated with an S100A antibody (1:100) at 4 °C in a humid environment overnight and then the goat anti-rabbit secondary antibody (1:200; ThermoFisher Scientific) was added for 1 h at room temperature. 4′,6-diamidino-2-phenylindole (DAPI) was used to stain the nucleus (Sigma-Aldrich), and the cells were imaged using a fluorescence microscope (Nikon, Tokyo, Japan).

### 2.9. Immunohistochemistry

The paraffin-embedded muscle sections were deparaffinized and hydrated with xylene (Junsei, Tokyo, Japan) and ethanol (Merck), respectively. Endogenous peroxidase activity was quenched with 0.3% H_2_O_2_/methanol, and these were then incubated with 1% normal goat serum. S100A antibody (1:100) was added, which was followed by incubation at 4 °C in a humid environment overnight with subsequent incubation with a horseradish peroxidase (HRP)-conjugated secondary antibody (1:100). The signals were detected by adding HRP-conjugated streptavidin (Vector, CA, USA). Nuclei of stained sections were stained with hematoxylin, and the sections were then dehydrated, mounted, and observed using a light microscope (Leica, Wetzlar, Germany).

## 3. Results

Table 1 shows the details of each system and durations of all-atoms unbiased MD simulations performed in the study. Each system was repeated two more times with consistent results, and the simulations were initiated with randomly selected initial velocities.

### 3.1. Stability Analysis of Each System

The root mean square deviation (RMSD) values of the backbone atoms (Table 1) are provided in the supporting material (supporting material S1). The RMSD values of each system increased at the beginning with respect to its native structure. In the apo-S100A1 system (black line), the values deviated from the native structure within ~5 ns of simulation and fluctuated until ~35 ns, and remained almost flat at the end of the simulation. The noted observed value on average was >0.39 nm. Yet, the initial larger RMSD values (>0.55 nm) until ~20 ns was noted in the holo-S100A1 system (red line). It was further observed that the domains of the S100A1 protein were significantly altered due to Ca^2+^ and that the alternations occurred significantly near the H3-EF site-H4 region. Although the holo-S100A1 system was simulated to a significant extent i.e., for 100 ns of simulation-1, the discrepancy from the initial structure within the first ~20 ns was sufficient to indicate that the S100A1 structures were substantially transformed at the H3-EF site-H4 region. In the next section, we elaborated on the RMSDs of the H3 and H4 regions independently. In the test of entry of free ions, the RMSD value (blue line) appeared to be stable and the difference of the RMSD from the native structure was noted to be ~0.1 nm. RMSD values form a plateau until the end of the simulation since ions in bulk were considered to reach the first salvation shell of protein after >10 ns simulation time. The average RMSD was noted to be ~0.22 nm in simulation-1. It shows a behavior similar to that of the holo-S100A1 system and is rooted by the effect of Ca^2+^.

The comparison of the root-mean-square fluctuation (RMSF), calculated for each trajectory, reveals approximately similar peak until the residue number ~40 (supporting material S2). After this, a single higher peak was found between residue numbers 40 and 60, where RMSF mean values were calculated as ~0.4 nm, which suggested a more flexible region. These noted flexibilities occurred due to conformational changes and this section was responsible mainly for H4. Nearly the same patterns of RMSF were found in two more recurrences of the simulations.

**Figure 2:**
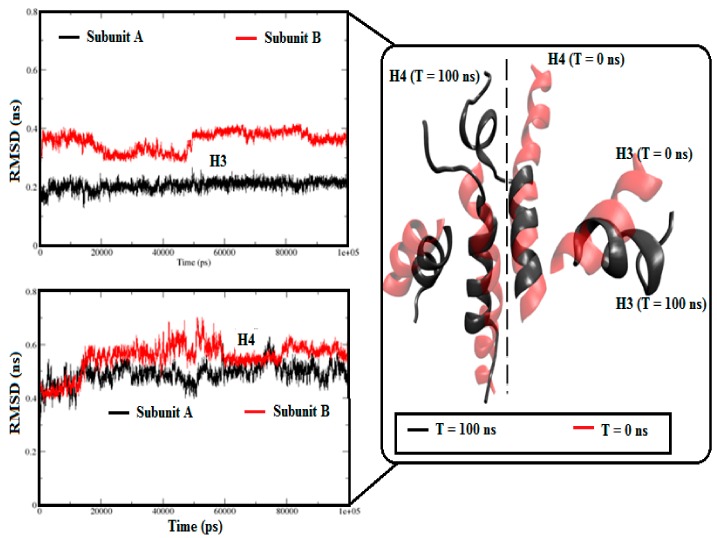
RMSD approximations of H3 and H4 domains in both subunits of the holo-S100A1 system. RMSDs of H3 and H4 domains of the two sides are represented in a red and black color code. A comparative superimposition between the structure of T = 0 ns (cartoon in red) and T = 100 ns (cartoon in black) of H3 and H4 of the two sides. Comprehensible structural transitions can be seen after simulation.

Considering little but significant RMSD variations in the holo-S100A1 system, we additionally calculated RMSDs for both sides along with the H3 and H4 domains (Figure 2). Figure 2 shows that the RMSDs of H3 and H4 in subunit A exhibited a fairly flat pattern, which revealed a reasonably stable helical domain. In contrast, the RMSDs of H3 and H4 in subunit B exhibited fairly uneven patterns and a significant departure from the native structure. The RMSD variations were due to the effect of the load of Ca^2+^ on the H3, EF site, and H4 topological regions. For comparison purposes, superimposition of the H3 and H4 domains of each subunit was further executed. Consequently, structural transitions were noted during the simulation. These observations revealed a conformational alteration in H3 and H4, which was more apparent in subunit B.

### 3.2. Effect of Ca^2+^ on the S100A1 Protein

**Figure 3:**
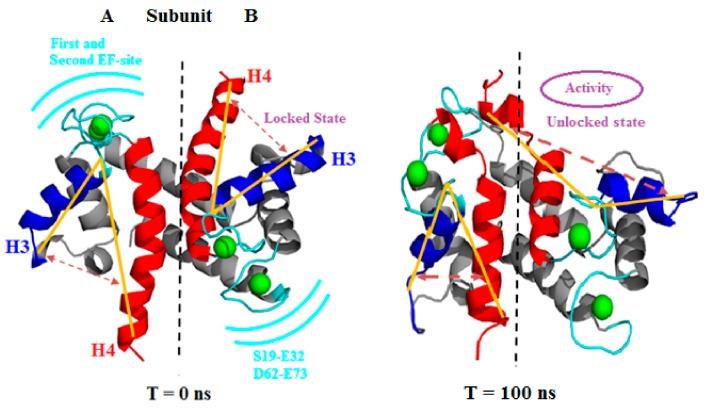
Visualization of first (T = 0 ns) and last (T = 100 ns) frame of the holo (PDB: 2LP3) S100A1 protein. The figure clearly shows the locked and unlocked states in both sides A and B. The yellow lines are drawn to show the expansion of the opening that leads to the unlocked state (hydrophobic patch) and activates the PPI. H3 and H4 of each subunit are colored in blue and red, respectively. The position of the first and second EF site are represented in a cyan color along with a residue range. Ca^2+^ are depicted as green balls.

Figure 3 shows the extensive conformational changes due to Ca^2+^, especially observed at the H3 site, EF site, and H4 domains of both subunits during the simulation. Eventually, both subunit transitions were originated independently.

#### 3.2.1. Angle Estimations between Helix-Loop-Helix Domains

The angle for each subunit was calculated at 10 ns, 40 ns, and 80/100 ns time frames for the apo and holo states. We have monitored the angle at every five levels of the time frame whereas time frames of 10 ns, 40 ns, and 80/100 ns were considered during the simulation. Table 2 shows the average angle value during the simulation, and, since S100A1 protein was characterized assymmetric, Cα atom of Lys56, Glu73, and Trp90 residues that represent three points of H3, EF site, and H4 domains were considered for angle calculations. The angle value was further averaged within each time frame of subunit A in the apo-S100A1 system. It was noted to be ~67.22°. Whereas, in subunit B, a variation difference of ~10° was observed, which eventually ends with approximately the same angle value of 66.94°. In addition, we observed significant increments in the angle at each further respective time frame, found between the H3-EF site-H4 domains of the holo state, which starts opening relatively faster than the apo state. The average angle of subunit A for each time frame was calculated as 72.59° and the difference between the 10 ns and 100 ns time frame was observed to be 14.29°. In subunit B, the average angle was calculated as 84.35° and the difference between 10 ns and 100 ns was noted to be 42.73°.

Angle calculations in the apo and holo states showed a vast increment, which was in strong agreement with the findings of previous studies [25,26]. Since the interhelical angles constantly increased, the standard deviation showed little fluctuations at the later stage of simulation (after 50 ns), as compared to the initial stage.

#### 3.2.2. Calculation of a Solvent Accessible Surface Area and Hydrogen Bonds for the Apo-State and Holo-State of the Protein

**Figure 4:**
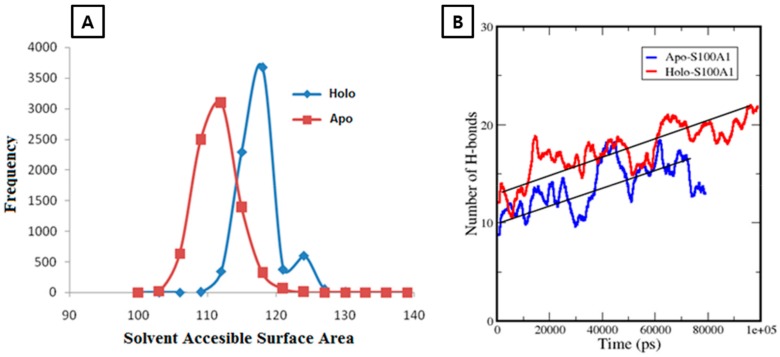
**A** represents the solvent accessible surface area (SASA) over the frequency distribution of apo-S100A1 and holo-S100A1. **B** shows H-bond changes in an apo (red) and holo (blue) state during simulation of 80 ns and 100 ns, respectively. Calculations take place between subunits **A** and **B** of each system separately at a cut-off of 0.35 nm. Straight black lines are drawn to depict the average of H-bonds of each system. **A** rise of ~10 H-bonds in holo-S100A1 revealed firmness of the system.

To deduce the conformational changes, SASA changes at each frequency point were monitored. In the apo-S100A1 system, an increment in SASA started at a value of ~102 nm^2^, and later reached ~112 nm^2^ at a maximum frequency point of ~3250 (Figure 4). From the highest point, a considerable decrease in SASA was observed. Eventually, aggregation of frequency points was observed at the end and a frequency distribution plot showed a Gaussian distribution, which normalized the frequency at each point. Three points in SASA were observed above the 1000 frequency. In case of the holo-S100A1 system, a larger difference was found in the parallel direction, from ~110 nm^2^ to 128 nm^2^. The significant increment in areas was accounted for by the presence of Ca^2+^. Although differences between each point in the apo and holo system cannot be uniquely attributed, the differences are, in any case, relatively small. However, two points in SASA were observed above 1000 frequency. It was clear that the rise in surface areas is rooted because of the Ca^2+^ bound to the EF site.

A considerable transition in H3 and H4 was an indication that there may be a possibility to form new bonds between both subunits. This motivated the calculation of H-bonds in each trajectory. Consequently, subunit A and B were compressed toward each other and, specifically, the H4 domain of one subunit opened and effectively moved outward in the direction of the opposite subunit. To quantize this observation, we decided to take the H4 domain of each subunit as two points to calculate H-bonds. Thus, the significant mobility of H4 of each subunit was a boost and caused the formation of approximately nine H-bonds at the end of the simulation. These revealed that an extensive interaction occurred between subunit A and B through the H4 domain. These interactions were expected for a stable architecture of S100A1 as a functional protein. However, in the apo state, only about four incremental H-bonds were observed during simulation, and, as expected, no considerable conformational changes occurred (Figure 4B).

#### 3.2.3. Temporal Distributions for Apo-State and Holo-State of the Protein

**Figure 5:**
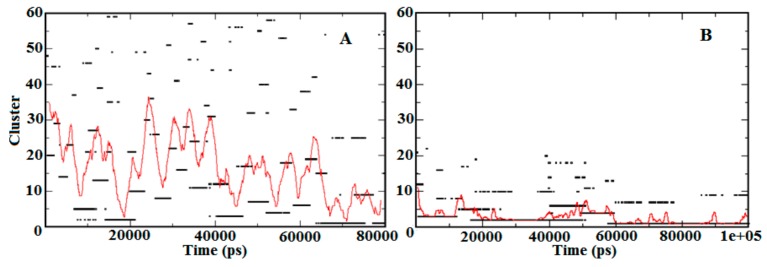
Temporal distribution of the apo-S100A1 and holo-S100A1 state. **A** denotes cluster distribution approximation of the apo-S100A1 protein, which demonstrates larger clusters by time, and **B** represents the holo-S100A1 (Ca^2+^ loaded) protein illustrating ~20 clusters noted until 60 ns and the remaining 10 clusters in the last 40 ns of simulation. The estimation of cluster analysis is computed using the cut-off value of 0.2 nm. The red trace represents the mean cluster value calculated for a 100 ps wide running time-window.

To refine the search for structural transitions that may be discriminated between the apo-S100A1 and holo-S100A1 proteins, trajectories 1 and 2 as listed in Table 1 were subjected to cluster analysis. Using a cut-off of 0.20 nm, the analysis produced about 60 clusters with sizes that were sufficient to convince statistical significance. The temporal distribution of the clusters in both states conferring to their size is shown in Figure 5. Figure 5A demonstrates the relative sizes of the clusters as calculated during simulations initiated with no Ca^2+^ in the subunit protein. Figure 5B presents a similar analysis for the simulations where the ion was initially located at the EF site. In the case of A, more than 60 clusters were found, which indicates less stability in the temporal distribution of the S100A1 structure. Furthermore, the distribution of clusters was somewhat noisy and the sequence of conformational changes were comparatively minor, which suggests a regular progress toward an unstable and inactive state of subunits A and B. The large dominating clusters are in rapid equilibrium with almost lesser lifetime structures. Temporal distribution shown in Figure 5B revealed the three largest clusters with a larger life span. The simulation time noted for all three representative structures was found to be from 0 ns to ~13ns, ~15 ns to ~50 ns, and ~60 ns to the end of the simulation. This less noisy and mostly rigid cluster distribution suggested stability in the representative structure of the largest cluster. Thus, the amplitude of the red trace indicates the extent to which the protein tends to sample low probability configurations. This scattering of the cluster’s popularity is a measure of fast structural transitions.

A comparative analysis between the demonstrative structures of the biggest clusters specifies that the variances between these are entirely at the EF site linking the helices H3 and H4. Examination of the average temporal distribution of the clusters (red line, Figure 5A,B) divulges that the large clusters dominate over long time stretches (10–40 ns) with brief disruptions of high probability conformations. This demonstrates that the system is in fast dynamic equilibrium toward stabilization (Figure 5B). Speciously, Ca^2+^ presence affects directly on the distribution function of the cluster.

### 3.3. Contact Map Analysis between Subunits along the Trajectory

Since the activity of a protein-mediated unlocked state takes place due to Ca^2+^, it involves the relative motion of the subunits, a process where the pattern of contact, by way of minimum distances between the domains of both subunits varies. Recently, in such studies, contact analysis was attempted to elucidate the domain-domain interaction during simulation [35]. To quantify these interactions, scrutinizing all the residues intricated in the contact between the suitable domains, with further approximation of the average (geometric) distance between them throughout a particular time frame along the trajectory was carried out. The interacting residues of the subunits A and B are marked on a vertical and horizontal axes, respectively. The colored boxes describe the structural features of each domain that interact with the respective domain of another subunit. The colored eclipse represents the significant contact distances made between the residues on the H1 site, the EF site, and H4 of both subunits of the protein.

**Figure 6:**
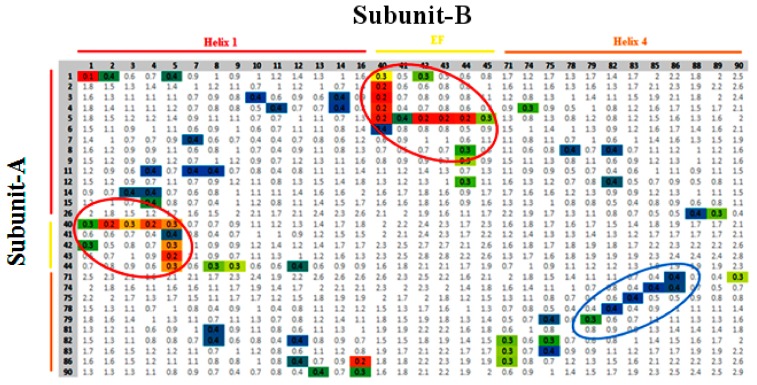
Contact analysis between adjacent domains of subunit A and B in the S100A1 protein during the simulation. The residues of subunit A are detailed in rows, while the subunit B residues are listed in columns. The pattern of contacts between the residues of H1, EF site, and H4, are colored in red, orange, and yellow bars, respectively. The colored pixels represent residues that are at a distance of less than 0.4 nm. The color code of separation distance, shown as mean distance, is blue, which represents a mean distance of more than 0.35 nm. Green represents a mean separation of less than 0.35 nm and more than 0.3 nm. Yellow represents a mean distance of less than 0.3 nm. Red and orange symbolize an average distance of 0.1 nm and 0.2 nm, respectively. The analysis was performed over the time frames of the largest clusters of unlocked and locked states. Red eclipse denotes all significant contacts made by the H1 and EF site of each subunit, whereas the blue eclipse defines the interaction between H4 of each subunit.

The number of contacts as calculated for the relaxed conformations of the unlocked state of holo-S100A1 protein revealed that the H1 site, the EF site, and H4 domains of each subunit were making contact during the simulation (Figure 6). The contacts between the subunits were a consequence of some structural deformations. The major deformation occurred because of the significant inclination of the EF site of subunit B toward H1 of the next subunit and vice versa. The residues Glu, Leu, Ser, Gly, and Phe (residue numbers 40–45) were recruited mainly to establish robust contacts. These contacts with distances mostly less than 0.3 nm between corresponding residues are depicted in color boxes within red eclipses. Distances between the EF site and H1 domain’s residues in each subunit retained their contacts during the simulation. Other residues in the EF site, mainly carboxylates, engaged in Ca^2+^ binding are illustrated in later sections. In the earlier section, we have confirmed that the H4 domain of subunit B shows enormous structural deformations, which leads to the unlocked state. The unlocked state assumed active conformational changes, which suggests that the bending of the helices can serve as enhancement of contacts with H4 of subunit A (blue eclipse, Figure 6). In this contact, mainly Ala, Leu, Thr, Val, Cys, and Asn residues of subunit B were enlisted, which effectively connected with residues Phe, Gln, Glu, Tyr, Val, Leu, and Ala of subunit A. The difference between the H4 domains appears to be an intensive translocation in the size and nature. These observations, together with some sort of major structural changes, indicate that the active-inactive transition is tied with the H1 site, the EF site, and H4 domains in both subunits.

### 3.4. Monitoring of Ca^2+^ Binding Residues

**Figure 7:**
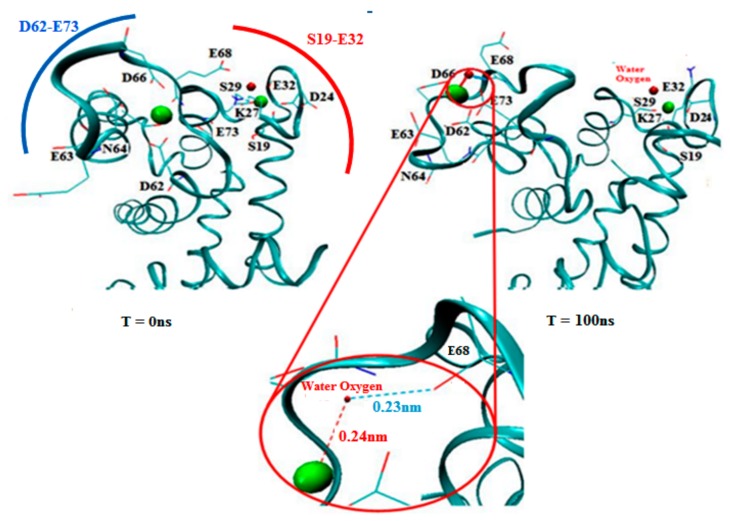
Visualization of subunit B of holo S100A1 after simulation. Residue atoms closer than 0.3 nm are denoted as a line (oxygen in red, carbon in cyan, and nitrogen in blue). The left frame at T = 0 depicts the inverted position of subunit B. Right frame B represents the propensities of binding residues with Ca^2+^ at T = 100 ns. A magnified view in the red circle denotes the H-bond formation where the oxygen atom of E68 forms a H-bond with Ca^2+^ via oxygen in water. Ca^2+^ are also shown (in CPK style) along with the relevant distances.

Since subunit B exhibited extensive conformational changes in the topology of the H3-EF site-H4 domains, we decided to visualize subunit B independently to illustrate the binding residues. Figure 7 shows a magnified view of subunit B of the S100A1 protein with a detailed view of the binding sites and two Ca^2+^ depicted, as reproduced from the crystallographic structure provided by Nowakowski et al. [26]. Carboxylate moieties of the EF site participate in the coordination with Ca^2+^ either directly or via a water molecule. Moieties closer than 0.3 nm to Ca^2+^ are considered to form the salvation shell of the ion. The hydrophobic patch generated a gap that occurred due to the hydrophobic residues lying on the H3-EF site H4 topology, which is in agreement with published data [13,19]. Within the EF site domain, it was revealed that moieties such as K and carboxylate D and E interacted mostly with Ca^2+^, where E73 was initially located detached from the EF site. However, during simulation, it was shifted toward the Ca^2+^ (Figure 7 rightmost). On the other hand, additional residues that were far from the EF site, like N64 and S29, became the probable accessible moieties during the simulation. Figure 7 clearly illustrates residues that interacted with each Ca^2+^ in the EF site. It was observed that the carboxylate residues perform rapid dynamics and move close to each Ca^2+^. Therefore, carboxylate residues and K27 serve as a preferred site for ~80% and ~20% of the simulation time, respectively. Single H-bonds were generated through a water molecule between E68 and a Ca^2+^, which secured the propensity of the ion toward the EF site residues.

Previously, it has been reported that the 14 residues between S19 to E32 were distinguished as the first EF region, whereas the second EF region is unique to the S100 family and related proteins, with 12 residues linking D62 and E73 [9]. Binding residues indicate that the initial ligands were consistent with the carboxylate oxygen atoms of D24, E32, E63, D66, E68, and E73 (EF site), but D66 and E68 were used to recruit crucial ligands. On the other hand, N64, S29, and K27 were also involved to some extent at the beginning of the simulation. In trajectory, a high time resolution trace that expands the passage between the sites is presented in Figure 7. Following this period, there were brief events (few ns long) where the carboxylate moieties E32, D66, and E68 participated as the next encounter moieties of the ion. At the end of the transition step, residues D24, E32, D66, and E68 were the dominant moieties for the ion. A minimum distance (less than 0.25 nm) was covered for the contact with D66. E68 shifted towards Ca^2+^. In addition, the red circle shows a magnified view of establishing a H-bond between the E68 residue and Ca^2+^ through an oxygen atom of the water molecule. However, the contact with D24, K27, and E32 still continued in the middle stage of the simulation, while, at this stage, N64 was not in contact any more. At the end stage, the interaction with the residue S29 was also established and the ion was anchored to subunit B. However, since the ion can build a strong interaction with S29 and restore a brief contact with K27, the system is in a rapid transition between equipotential conformations (Figure 7).

### 3.5. Entry of Free Ca^2+^ into the Bulk of the Apo-State Protein

**Figure 8:**
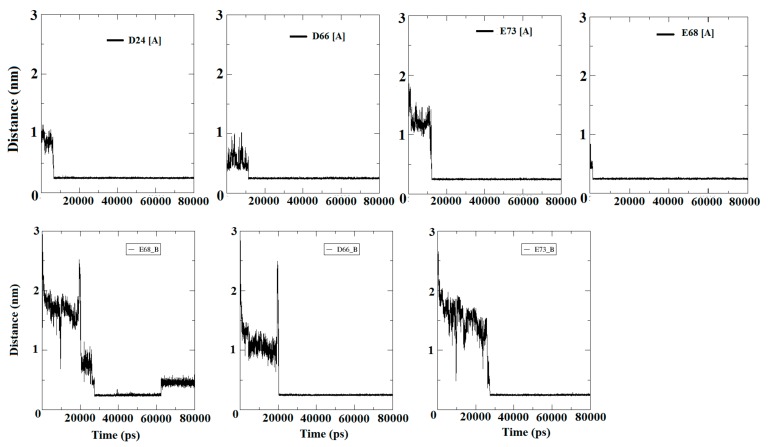
Distance calculations from Ca^2+^ to specific residues listed in the above analysis. Residues and its identifiers given along with subunits A and B demonstrated four and three carboxylate moieties in subunit A and B, respectively. Each Ca^2+^ reaches a significant distance between ~5 ns to 25 ns simulation time and maintains equivalence throughout.

The mechanism, by which Ca^2+^ travel to the surface of the protein and find the EF site domain, was investigated by the present experiment. Moreover, specific binding residues were also emphasized while examining first and second encounters of ions. In addition, total residence time was also provided to evaluate the binding robustness. The four Ca^2+^ were added to the water box of the apo-S100A1 structure, which was initially in bulk, and found their positions randomly. Entry led to an average frequency of the encounter, where the Ca^2+^ approached the solvating shell and found preferable carboxylate moieties. Residues that were identified in the first and second encounters of a Ca^2+^ were mainly D24, E32, D66, E68, and E73. It was observed that all these identified residues belong to the EF site domain. Due to these residues, the EF site domain was established as a highly negative region of the structure, where ions appear to be resolved. In the first observation, to quantize this event, we approximated the minimum distance between the identified residue and, collectively, all Ca^2+^ (Figure 8).

Figure 8 confirms that the Ca^2+^ were in bulk initially and discovered crucial surface residues within the time period from ~6 ns to ~28 ns. D24, D66, E68, and E73 were identified as crucial moieties, which acquired enormous propensities in the direction of Ca^2+^. These residues of subunit A were reached with an appropriate distance of <0.30 nm (solvation shell) in ~6–10 ns, and remained horizontal at the same distance until the end of simulation. D66 and E73 of subunit B were identified as crucial residues that maintain a distance of approximately 0.25 nm with Ca^2+^ at simulation time of approximately 20 ns and 26 ns for D66 and E73, respectively. The conclusions drawn for this experiment of entry of Ca^2+^ in bulk were supposed to be significant for ion circulation on the surface of the EF site. In addition, all four Ca^2+^ were adsorbed and started disappearing from the bulk very rapidly between ~10 ns to ~20 ns. In case of the E68 residue, the ions achieved appropriate distances at the very beginning of the simulation. Such an arrest of a Ca^2+^ had been observed on EF sites by reason of a local attractive electrostatic potential [36,37]. A comparison of the binding phenomenon using the crystal structure revealed that the ions accumulated on the EF site and also on residues of the adjacent domain to facilitate their contribution in the role of S100A1 activity. Remarkably, once the ion is made obtainable by the protein shell, its entry to the EF site is improved. The path between the two surroundings requires no more than a few ns and is guided by reducing the water molecules in the solvation shell.

To gain insights into the free entry of ions on the EF site and its adjacent domain residues along with conformational changes, angles were again estimated by indexing the same residue numbers guided by the yellow line in Figure 3. On comparing with holo S100A1, some significant elevation in angles during the simulation was observed. Table 3 reports the angle estimations in various simulation time frames. The time frame considered was 10 ns, 40 ns, and 80 ns, where a substantial increase in angle values was noted. An angle difference of 45.46° was noted for subunit A, which possessed a high resemblance with the holo-S100A1 system. Whereas, in subunit B, the angle elevation noted was relatively slow and the difference in time frames between 10 ns and 80 ns was found to be 14.67°. Thus, due to the ion encounters, the angle values were increased, which corroborated the resemblance with the holo-S100A1 system.

#### 3.5.1. Encounter of Ca^2+^

Table 4 illustrates the Ca^2+^ encounters and binding time on residual atoms. The binding time of Ca^2+^ is the contact time at which all possible moieties possess distances less than 0.5 nm. Mostly carboxylate moieties, D24, D66, E68, and E73, were identified in each subunit by means of high residence time with Ca^2+^. The residence time explains the total staying time of ions on the residues after the first and second encounters. Subunit A of the S100A1 protein exhibited residence time rather than Subunit B. This study revealed the motion of the Ca^2+^ and binding robustness of ions on the EF site residue. The total residence time was calculated using the following simple approximations. We have treated all the possibilities of the ion encounter on a residue. The maximum two encounters were obtained during the simulation time of 80 ns.

If the first encounter was found:

TRT = (80,000 − FET)(1)

If the second encounter was found:

TRT = (80,000 − SET)(2)

If the first and second encounters were found at different time intervals:

TRT = (SET − FET) + (80,000 − SET)(3)

where,

TRT, FET, and SET stand for total residence time, time of first encounter, and time of second encounter, respectively.

The accumulation of Ca^2+^ on the EF site at this location was retained for significantly many nanoseconds. Such interactions of an ion had been observed for defined sites on the surface of the protein where there is a local attractive electrostatic potential. According to this, the interaction of ions near the EF site provides a reservoir of the ions, which facilitates its participation in the functioning of the protein. Once the ion is made available, its entry to the binding site is enhanced.

#### 3.5.2. Ion Hydration and Dynamics of Water Molecules

**Figure 9:**
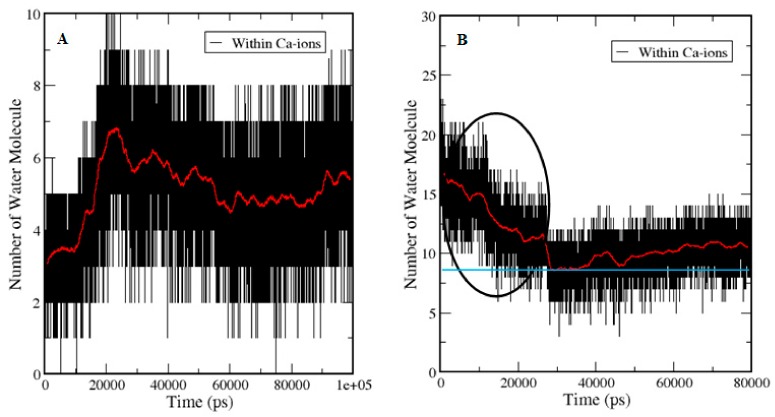
Hydration of Ca^2+^ and execution of water dynamics. **A** represents the calculation of water molecules in the holo-S100A1 system during simulation and B denotes the system of free entry of Ca^2+^ into the bulk. Red lines show the average number of water molecules around Ca^2+^ ions. The blue line in frame **B** indicates the mean point of the lowest number of water molecules. At the beginning of simulation, elevation in the number of water molecules is shown in A, whereas, in fB, a drop in water molecules is noted, revealing that the Ca^2+^ are approaching the surface of protein (black circle). However, after contacting the blue line, an elevation is noted similar to A, which suggests that a similar behavior occurred in holo S100A1.

The temporal distribution of the number of water molecules within the solvation shell, as calculated for the holo-state trajectories and trajectory of free entry of Ca^2+^, is presented in Figure 9. Calcium-binding sites per protein molecule with a dissociation constant (Kd) is in the range of 61 ± 4 μM to 210 ± 22 μM [38]. Therefore, the rate-limiting step of the ion entry is its partial desolvation event. All-atom unbiased molecular dynamic simulations allow a direct counting of the water molecules that were within the solvation shell of the ion as well as the dwell time of water molecules within this range.

In the holo state crystal structure, the ion was equally located on both subunits of the protein. During simulation, there was a fast exchange of its solvation water from the bulk. It was observed that the ions on the subunit of the protein were fully solvated and, thus, this event was calculated by selecting water molecules within the solvation shell of Ca^2+^, which traced their distance from the ion as a function of time (Figure 9A). The trace in Figure 9A demonstrates how water molecules approached from the bulk, and within ~20 ns, water molecules traveled into the solvation shell of the ion. The water molecules in the immediate vicinity of the ion are freely alternate within ~0.25 nm.

While, in Figure 9B, the ion is in bulk water. It was solvated by 15–18 water molecules in accordance with the published data [39]. Ions started approaching toward the salvation shell when the number of solvating water molecules was decreasing due to sharing of the surface area of the ions with carboxylate and carbonyl oxygen atoms (up to 0.25 nm) at the EF site. The lowest number of water molecules (blue line touches the red line) was reported at ~22 ns, which then started increasing, and remained elevated until the end of the simulation. In this case, the number of water molecules was decreasing and remained between 11–8 molecules. When the ion is not fully hydrated, the free coordinates were taken up by the moieties of the protein.

### 3.6. S100A Expression in Myoblast Differentiation and Muscle Regeneration

**Figure 10:**
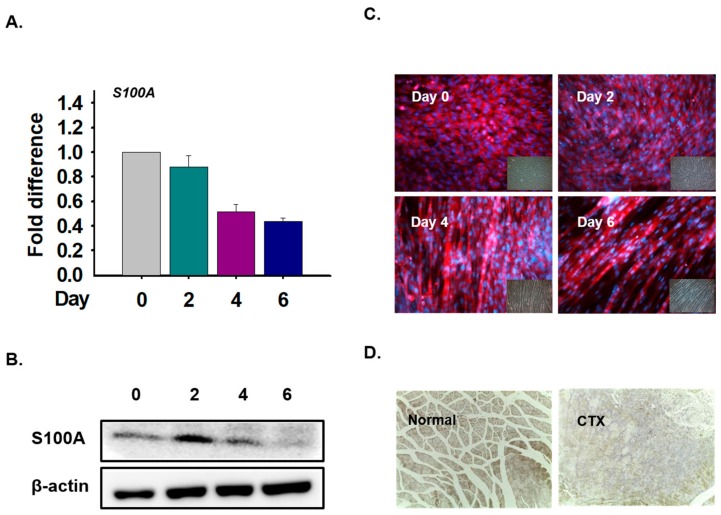
S100A1 expression in myoblast differentiation and muscle regeneration. C2C12 cells were cultured with 2% FBS for 0, 2, 4, and 6 days. mRNA protein expression was analyzed using real-time RT-PCR, Western blot, and immunocytochemical techniques. (**A**) mRNA expression using real-time RT-PCR. (**B**) and (**C**) S100A1 protein expression using Western blot and immunocytochemical techniques. CTX was injected in the GAS muscle and maintained for 7 days. (**D**) S100A1 protein expression in normal and CTX-injected muscles.

To investigate the function of the S100A1 protein in myoblast differentiation, C2C12 cells were incubated with differentiation media for 0, 2, 4, and 6 days. The expression of S100A1 mRNA decreased with differentiation time (Figure 10A). Protein expression was analyzed with Western blot and immunocytochemical techniques. The data showed that expression was detected in the cytoplasm and that protein levels were slightly increased in Day 2 cells (Figure 10B,C). In addition, S100A1 protein expression was observed in normal and CTX-injected muscles and showed that expression in normal muscles was higher than in CTX-injected muscles (Figure 10D). Thus, these data show that S100A1 expression is not significantly associated with inducing myoblast differentiation and muscle regeneration.

## 4. Discussion

The findings of a recent experimental study suggest that the S100 protein family can serve as diagnostic markers in neuronal and inflammatory disorders, myopathies, cancer, and many other diseases [40]. Recently, S100A protein partners were also discussed at a molecular level in normal and affected tissues [41]. Previously, genome and proteome level studies revealed specific Ca^2+^ binding proteins and highlighted their importance with respect to medical relevance [42,43]. Earlier, nuclear magnetic resonance studies have exposed atomic-resolution details of the S100A1 conformations in the apo-S100A1 [25,44], holo-S100A1 [11,25], and target protein-bound states [13,45].

During molecular dynamics simulation, the S100A1 protein responded mostly on the EF site of subunit B than subunit A. However, conformational changes are initiated simultaneously on both subunits (Figure 2). All the trajectories underwent stability checks, and each trajectory was found to be stable (Supplementary Materials S1 and S2). For securing convergence, the temporal distribution of clusters was calculated, which was characterized by a sequence of conformations with each persisting for longer lifetimes. This suggests a stable distribution of holo S100A1 (Figure 5). These distributions are very similar to those found in our previous study [46]. Earlier, several studies of PPI were analyzed through contact analysis [35], which contributes to the understanding of residue to residue distance analysis during this period (Figure 6). The contact map revealed vital contacts in diverse distance values between components, which illustrates a close agreement with appropriate deformations. This leads to protein activity.

The entry of four Ca^2+^ into the bulk provided an acquaintance. First, the effect of Ca^2+^ travelled toward the surface of the protein. Second, conformational dynamics, which lead to Ca^2+^ propagation, was extensively illustrated, and, third, the examination of Ca^2+^ translocation as well as reading the mobility by the entry of ions in the bulk was shown (Figure 8). Due to contact with Ca^2+^, significant conformational changes were observed, which was reported in previous studies [47]. Water dynamic approximation during a binding event revealed that the volume of water was noted to be significantly less during the corresponding simulation time, which assumed that Ca^2+^ are appreciably traveling toward the protein surface (Table 4, Figure 9).

Previously, the study of Ca^2+^ at low and high concentrations suggested binding events of carboxylate with the ions, given that saturation of the EF site requires Ca^2+^ concentrations of 100–10,000 micromolar [48,49]. The ion was first ligated on the EF site by the carboxylate of D66, E68, and a single water molecule formed a bridge between the ion and the carboxylate of E68. Second, an intermediate state where the solvation shells of the ion included both the carbonyl residue K27 and the carboxylates of D24 and E73 with no water molecule between the ion and the carboxylate moieties. Third, and the most popular residues of the Ca^2+^, for the crystal structure of S100A1, were found to be the carboxylate of D66 and E68.

The large-scale study of the human transcriptome established that the human heart is the leading location of S100A1. The expression of S100A1 progressively increases throughout cardiac development in mice and reaches a plateau in the ventricular myocardium in the postnatal state [3,50]. In a study, although the S100A1 protein was not uniformly expressed in the heart of an adult rat, nonetheless, it exhibited higher levels of mRNA and protein in the left ventricle but showed lower concentrations inside the right ventricle and atria [51]. There are several pieces of evidence to show a crucial role of S100A1 in fine-tuning skeletal muscle Ca^2+^ release. S100A1 binds to ryanodine receptor type-1 and plays an imperative role in the excitation-contraction coupling (ECC) process of skeletal muscle [52,53]. In the ECC of skeletal muscle, the sarcolemma depolarizes by means of action potentials to release Ca^2+^ from the sarcoplasmic reticulum [54]. The study of S100A in different stages of myogenesis and in muscle regeneration was not yet reported from scientific literature. In this study, we found that the S100A1 gene decreased during the myogenic differentiation and muscle regeneration process. In a study by Mori et al., mice lacking S100A1 as well as S100B genes exhibited normal skeletal growth from an embryonic adulthood stage [55].

## 5. Conclusions

The crystallization of the protein in the presence of ions could not reveal a structural feature clear enough to account for conformational changes-mediated functions. A clear outcome of the simulations is that the holo protein grossly deviates from its crystal structure. The study provides an explanation for access to the Ca^2+^ preferably by the EF site. Reflection of the protein function suggests that the enhancement of catalysis by the ion leads to stabilization of the conformation of the protein. The entry of free Ca^2+^ into the bulk explained the binding events. In addition, ion movement and water dynamics supported the observation of ion contact. These findings are valuable for understanding the function of the broad family S100 and the results may also be supportive of the study of Ca^2+^-mediated PPI. For instance, because of the role of the ion as a key regulator of cardiac function, cardiomyopathies, and heart failure, S100A1-based gene therapy may be developed for clinical trials. Additionally, our results demonstrate the downregulated expression of S100A1 in the differentiation steps of myogenesis with no significant effect in the muscle regeneration process.

## Figures and Tables

**Table 1:** Details of the systems and the duration of simulations and recurrence.

Serial No	System	PDB id	Number of Ca^2+^ in Solvent Phase	Duration of Simulations (ns)	
				Simulation-1	Simulation-2
1.	**Apo-S100A1**	2LLU	nil	80	0
2.	**Holo-S100A1**	2LP3	4	100	80
3.	**Entry of free Ca^2+^**	2LLU	4	80	80

**Table 2:** Angle estimations between H3-EF site-H4 topology at various simulation time intervals for each subunit. Angles are calculated based on the yellow line in Figure 3. More than 10° angle differences in apo and holo states are depicted in bold and italic fonts.

System	Subunit A			Subunit B		
	10 ns	40 ns	80 ns/100 ns	10 ns	40 ns	80 ns/100 ns
**Apo-S100A1**	64.34 +/− 5.82	68.71 +/− 5.18	***69.82 +/− 4.72***	***46.42 +/− 6.86***	***54.89 +/− 13.34***	***66.94 +/− 17.02***
**Holo-S100A1**	65.65 +/−5.12	72.19 +/− 7.59	***79.94 +/− 9.97***	***66.40 +/− 1.64***	***79.66 +/− 6.58***	***106.13 +/− 11.99***

**Table 3:** The experiment of entry of four Ca^2+^ from the bulk for each simulation is approximated between the H3 site, the EF site, and H4 topology in the respective time intervals for each subunit. Angles are calculated according to the same residues, as directed by the yellow line of Figure 3.

System	Time		
**Entry of Ca^2+^-S100A1**	10 ns	40 ns	80 ns
**Subunit A**	66.75 +/− 8.23	77.50 +/− 5.97	80.31 +/− 5.84
**Subunit B**	47.34 +/− 5.96	42.30 +/− 5.93	62.01 +/− 8.76

**Table 4:** The estimation of the total residence time of a Ca^2+^ on the corresponding residue whose distance is less than 0.5 nm. The time of primary and secondary encounters of Ca^2+^ on the residue is counted. The encounters in bold indicate significant residence time.

Residues	Ca^2+^ Encounter on Subunit A (Time in ps)		TRT (ps)	Ca^2+^ Encounter on Subunit B (Time in ps)		TRT (ps)
	FET	SET		FET	SET	
**D24**	**7948**	**0**	**72,057**	**9411**	**0**	**33,566**
E32	0	0	0	0	0	0
K27	58,594	77,385	22,856	0	0	0
D62	0	0	0	588	0	19,118
**D66**	**11,028**	**0**	**68,975**	**20,135**		**59,865**
**E68**	**280**	**0**	**79,720**	**22,750**	**0**	**40,004**
**E73**	**588**	**0**	**79,412**	**28,882**	**0**	**51,118**
S29	3202	11,046	5394	21,156	25,750	1548
N64	10,046	0	1980	0	0	0
S19	0	0	0	0	0	0
E63	11,699	41,928	15,686	1078	0	18,138

## Supplementary Materials

The following are available online at https://www.mdpi.com/2073-4409/9/1/181/s1, Figure S1: Approximations of Root mean square deviation (RMSD) of each systems of both simulation. RMSDs of Apo-crystal (black), Holo-S100A1 (red) and experiment of free entry of Ca^2+^ ions into bulk (blue), are showing a plateau after half of the simulation, Figure S2: Calculations of Root mean square fluctuation (RMSF) of each system which is listed in Table 1. RMSFs in each system demonstrating same pattern till residue number ~40. Panel **A** represents RMSF of apo-S100A1 during simulations. Panel **B** depicts the RMSF of holo-S100A1 and Panel **C** corresponds to system of free entry of Ca-ions in the bulk respectively.
