# Supplementary material for: Understanding Calcium-Dependent Conformational Changes in S100A1 Protein: A Combination of Molecular Dynamics and Gene Expression Study in Skeletal Muscle
Source: Cells. 2020 Jan 10;9(1):181. doi: 10.3390/cells9010181 (PMC7016722; doi:10.3390/cells9010181)
Supplement: Supplementary file 1 [file cells-09-00181-s001.pdf]

Additional Information

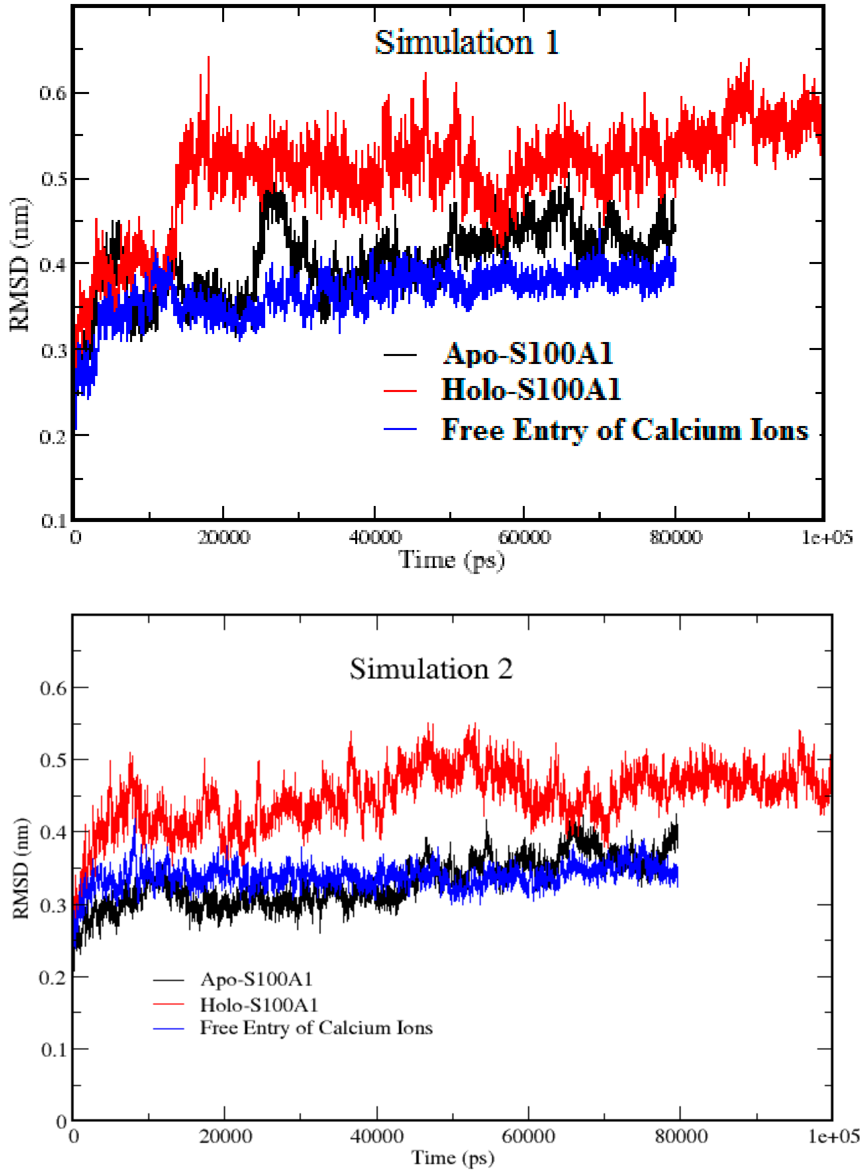

Figure S1. Approximations of Root mean square deviation (RMSD) of each systems of both simulation. RMSDs of Apo-crystal (black), Holo-S100A1 (red) and experiment of free entry of Ca^{2+} ions into bulk (blue), are showing a plateau after half of the simulation.

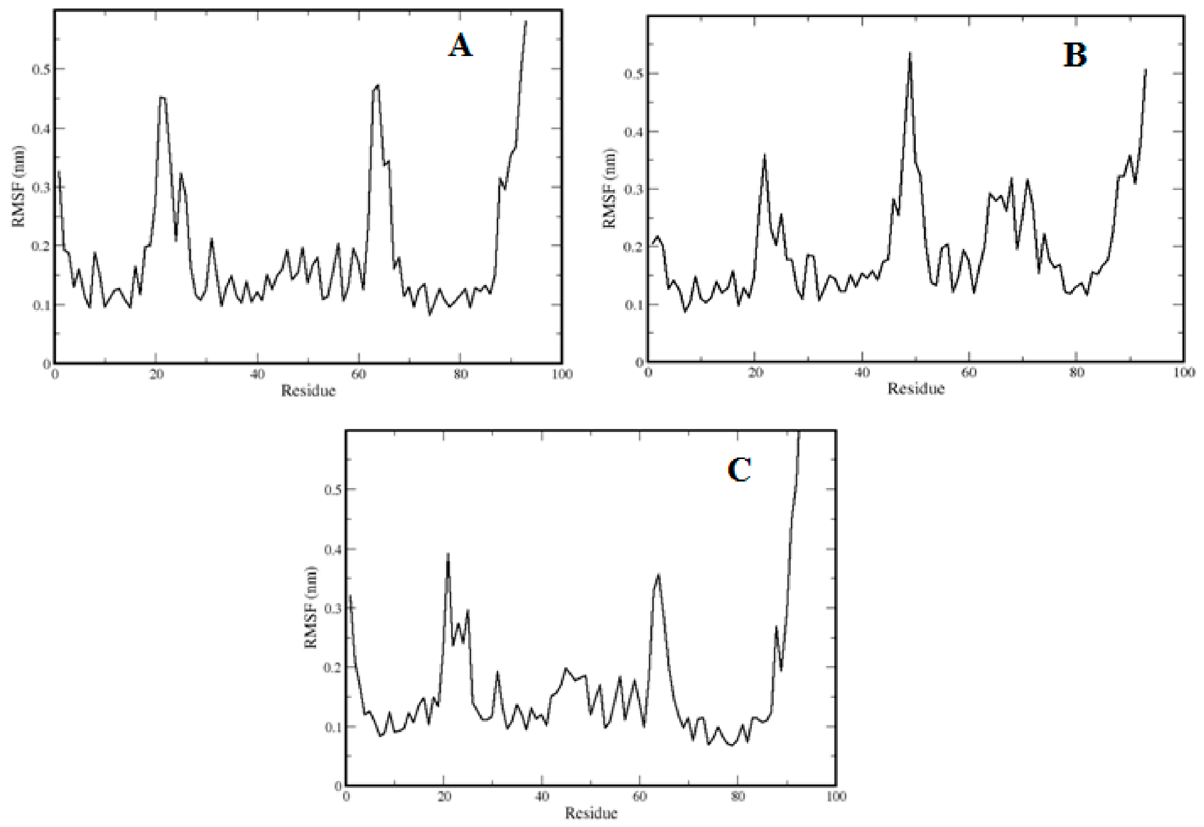

Figure S2. Calculations of Root mean square fluctuation (RMSF) of each system which is listed in Table 1. RMSFs in each system demonstrating same pattern till residue number ~40. Panel **A** represents RMSF of apo-S100A1 during simulations. Panel **B** depicts the RMSF of holo-S100A1 and Panel **C** corresponds to system of free entry of Ca-ions in the bulk respectively.
